# Single acute stress-induced progesterone and ovariectomy alter cardiomyocyte contractile function in female rats

**DOI:** 10.3325/cmj.2014.55.239

**Published:** 2014-06

**Authors:** Judit Kalász, Enikő Pásztor Tóth, Beáta Bódi, Miklós Fagyas, Attila Tóth, Bhattoa Harjit Pal, Sándor G. Vári, Marta Balog, Senka Blažetić, Marija Heffer, Zoltán Papp, Attila Borbély

**Affiliations:** 1 University of Debrecen, Faculty of Medicine, Institute of Cardiology, Division of Clinical Physiology, Debrecen, Hungary; 2 University of Debrecen, Faculty of Medicine, Department of Laboratory Medicine, Debrecen, Hungary; 3 Cedars-Sinai Medical Center, International Research and Innovation Management Program, Los Angeles, CA, USA; 4 J. J. Strossmayer University of Osijek, School of Medicine, Department of Medical Biology, Osijek, Croatia

## Abstract

**Aim:**

To assess how ovarian-derived sex hormones (in particular progesterone) modify the effects of single acute stress on the mechanical and biochemical properties of left ventricular cardiomyocytes in the rat.

**Methods:**

Non-ovariectomized (control, n = 8) and ovariectomized (OVX, n = 8) female rats were kept under normal conditions or were exposed to stress (control-S, n = 8 and OVX-S, n = 8). Serum progesterone levels were measured using a chemiluminescent immunoassay. Left ventricular myocardial samples were used for isometric force measurements and protein analysis. Ca^2+^-dependent active force (F_active_), Ca^2+^-independent passive force (F_passive_), and Ca^2+^-sensitivity of force production were determined in single, mechanically isolated, permeabilized cardiomyocytes. Stress- and ovariectomy-induced alterations in myofilament proteins (myosin-binding protein C [MyBP-C], troponin I [TnI], and titin) were analyzed by sodium dodecyl sulfate gel electrophoresis using protein and phosphoprotein stainings.

**Results:**

Serum progesterone levels were significantly increased in stressed rats (control-S, 35.6 ± 4.8 ng/mL and OVX-S, 21.9 ± 4.0 ng/mL) compared to control (10 ± 2.9 ng/mL) and OVX (2.8 ± 0.5 ng/mL) groups. F_active_ was higher in the OVX groups (OVX, 25.9 ± 3.4 kN/m^2^ and OVX-S, 26.3 ± 3.0 kN/m^2^) than in control groups (control, 16.4 ± 1.2 kN/m^2^ and control-S, 14.4 ± 0.9 kN/m^2^). Regarding the potential molecular mechanisms, F_active_ correlated with MyBP-C phosphorylation, while myofilament Ca^2+^-sensitivity inversely correlated with serum progesterone levels when the mean values were plotted for all animal groups. F_passive_ was unaffected by any treatment.

C**onclusion** Stress increases ovary-independent synthesis and release of progesterone, which may regulate Ca^2+^-sensitivity of force production in left ventricular cardiomyocytes. Stress and female hormones differently alter Ca^2+^-dependent cardiomyocyte contractile force production, which may have pathophysiological importance during stress conditions affecting postmenopausal women.

The relation between stress, gender, and cardiovascular diseases is well established (1-4). Some of the known risk factors for cardiovascular disease such as smoking, unhealthy diet, and behavioral and psychosocial stress have deleterious effects on the cardiovascular system via activation of the sympathetic nervous system and the hypothalamic-pituitary-adrenal (HPA) axis (5-8). Acute restraint stress is a preferred and widely used method to induce physical stress in animal models (9). Moreover, restraint and immobilization are important as models for psychological stress, which was shown to adversely affect ovarian function (10) and to play a pivotal role in the pathomechmanism of Takotsubo (stress) cardiomyopathy in postmenopausal women (11).

Gender is a very important factor in the development of cardiovascular diseases. Premenopausal women have better lipid profile, endothelial function (12), and a lower risk to develop coronary artery disease and myocardial infarction (MI) than men. These advantages of female gender, however, are abolished after menopause, which is associated with increased prevalence of left ventricular (LV) hypertrophy, decreased LV ejection fraction, and LV contractility (13). One of the explanations for the distinct myocardial responses is the cardioprotective effect of female sex hormones (eg, estrogens) (14,15).

Progesterone performs several actions on the heart: it exerts an antiarrhythmic effect by accelerating cardiac repolarization (16) and has a preventive role in ischemia-reperfusion injury via reducing inflammatory response (17). It has been shown to inhibit cardiomyocyte apoptosis (18), induce vasodilation, and reduce blood pressure via increasing nitric oxide (NO) levels in normotensive and hypertensive patients (19). Importantly, progesterone is produced by the both ovaries and the adrenal gland: Moreover, the adrenal progesterone content is similar or even larger than that in the ovaries (20). Adrenal progesterone production and secretion increase along with corticosterone regardless of gender and estradiol under stress conditions (21). Progesterone, being an indirect precursor of cortisol (22), increases in response to adrenocorticotrophic hormone (ACTH) stimulation (23).

In the heart, there are multiple estrogen hormone receptor types (24). The expression of aromatase in the heart suggests that estrogen may be synthesized also within the cardiomyocyte to exert autocrine/paracrine actions (25). Myocyte contractility seems to be modulated by systemic estrogen levels and altered in cardiomyocytes derived from ovariectomized (OVX) rats (26). In particular, myofilament Ca^2+^-sensitivity is increased in isolated myofibrillar preparations from OVX rats, and restored to the basal levels with estrogen supplementation (27,28).

Activation of the sympathetic nervous system plays a central role in the regulation of cardiomyocyte contractile function and myofilament Ca^2+^-sensitivity through beta-adrenergic receptor stimulation, activating the protein kinase A (PKA). PKA-mediated phosphorylation of Ca^2+^-handling and myofilament proteins (myosin binding protein-C [MyBP-C], troponin I [TnI], titin) were shown to alter cardiomyocyte contractile function (29,30). It has been suggested that female cardiomyocytes operate at lower levels of intracellular Ca^2+^ than those of males, particularly under inotropic conditions (31). This difference in Ca^2+^ homeostasis may be related to the fact that estrogen suppresses the L-type Ca^2+^ current (32,33) and may reduce the amount of Ca^2+^ released from the sarcoplasmic reticulum (SR) (34), which was shown to be larger in myocytes from OVX rats (35). Not only cardiomyocyte contraction, but relaxation may also be affected by estrogen via altered Ca^2+^ re-uptake into the SR and modified Ca^2+^ efflux via increased sarcolemmal Na^+^/Ca^2+^ exchange (36). Interestingly, despite similar SR Ca^2+^ content in males and females (37), studies using OVX models report conflicting results concerning changes in the expression and activity of the SR Ca^2+^-ATPase and its regulator protein phospholamban (38-41). Much less is known about the possible effect of progesterone on cardiomyocyte contractile function. We hypothesized that progesterone affected force production of single isolated cardiomyocytes. Therefore, in the present study we aimed to investigate how sex hormones (particularly progesterone) and single acute restraint stress altered cardiomyocyte contractile function and to identify the consequent posttranslational myofilament protein modifications in OVX rats.

## Methods

### Animals

The study was conducted using female Sprague-Dawley rats (n = 32) at the Department of Medical Biology of J. J. Strossmayer University of Osijek, School of Medicine (Osijek, Croatia) between June and September of 2013. Sixteen rats were ovariectomized at the age of 12 weeks. The ovariectomy was performed according to the protocol HUS-QREC-PRD-932 (Issue: 01, Revision 03). The anesthetized rat was placed in ventral recumbency with tail toward the surgeon. Following shaving and swabbing of the dorsal mid-lumbar area, a 2-3 cm dorsal midline skin incision was made halfway between the caudal edge of the ribcage and the base of the tail. Thereafter, a single incision 5.5-10 mm long was made into the muscle wall on both the right and left sides, and the ovaries and the oviducts were exteriorized through the muscle wall. A hemostat was clamped around the uterine vasculature between the oviduct and uterus. Each ovary and part of the oviduct was removed with single cuts through the oviducts near the ovary. The remaining tissue was replaced into the peritoneal cavity. The muscle incision was not sutured. Age-matched not operated female Sprague-Dawley rats served as controls (n = 16). Animals were housed in standard cages at room temperature with natural day and night exchange. Standard laboratory food and tap water were available *ad libitum*. At the age of 28 weeks, 8 control and 8 OVX rats were submitted to cold restraint stress: rats were placed and closed in a metal tube with a diameter that did not allow them to move or turn around. The animals were kept under these conditions in a cold room at +4°C for 1 hour (control-S and OVX-S groups). Immediately after the stress, the rats were anesthetized using combined inhalation of isoflurane (Forane^®^, Abbott Laboratories Ltd, Queenborough, United Kingdom) in a glass chamber and intramuscular administration of ketamine (Ketanest^®^S, Pfizer Corporation, Wien, Austria, 30 mg/kg body weight) and then sacrificed. Blood was drawn from the heart for plasma measurements. Thereafter, the left ventricles were cut and placed immediately into liquid nitrogen and stored at -80°C. The study protocol was approved by the Ethics Committee of the Osijek University School of Medicine. The heart tissue samples were transferred according to the regulations stated in the official material transfer agreement between the School of Medicine Osijek and the University of Debrecen (DETTI/22-3/2013).

### Determination of serum estrogen and progesterone levels

Estradiol was measured by a chemiluminescent immunoassay (Roche Diagnostics GmbH, Mannheim, Germany) using a specific polyclonal antibody against estradiol. The endogenous estradiol is released from the serum by mesterolone, which competes for the binding sites located on the biotinylated antibodies with estradiol derivative labeled with rutenium complex. The inter-assay coefficient of variation (CV) was <7% (lower detection limit: 4.9 pg/mL, upper detection limit: 4.3 ng/mL). Progesterone was measured using a chemiluminescent immunoassay (Roche Diagnostics GmbH) by using a specific monoclonal antibody against progesterone. The endogenous progesterone is released from the serum by danazol, which competes for the binding sites located on the biotinylated antibodies with progesterone derivative labeled with rutenium complex. The inter-assay CV was <5% (lower detection limit: 29.9 pg/mL, upper detection limit: 60.1 ng/mL). Serum glucose concentration was measured by an enzymatic, photometric method (Roche Diagnostics GmbH). As a first step, glucose-6-phosphate is formed from glucose and adenosine triphosphate catalyzed by hexokinase. In the presence of nicotinamide adenine dinucleotide phosphate (NADP) the glucose-6-phosphate is oxidized to gluconate-6-phosphate. The rate of formation of NADPH correlates with the glucose concentration and can be measured using photometry. Serum cholesterol was determined by an enzymatic, colorimetric method (Roche Diagnostics GmbH). Formation of H_2_O_2_ – generated during the oxidation of the cholesterol by cholesterol oxidase – leads to the generation of red quinonimine stain from 4-aminophenazone and phenol. The intensity of this red stain correlates with the concentration of cholesterol.

### Measurement of cardiomyocyte mechanical parameters

Force measurements were performed in single, isolated cardiomyocytes derived from left ventricular (LV) rat myocardium, as described previously (42). Briefly, samples were first defrosted in isolating solution (containing 100 mM KCl, 2 mM EGTA, 1 mM MgCl_2_, 4 mM Na_2_ATP, 10 mM imidazol; 40 µM leupeptin, 10 µM E64, pH 7.0), then mechanically disrupted and treated with isolating solution supplemented with 0.5% Triton-X-100 to solubilize the membranes and membrane-associated structures. Thereafter, single cardiomyocytes were mounted between a force transducer and an electromagnetic motor with silicone adhesive. Sarcomere length was adjusted to 2.3 µm. Relaxing (containing 37.11 mM KCl, 10 mM BES, 6.41 mM MgCl_2,_ 7 mM EGTA, 6.94 mM Na_2_ATP, 15 mM Na_2_CrP, 40 µM leupeptin, 10 µM E64; pH 7.2) and activating (containing 37.34 mM KCl, 10 mM BES, 6.24 mM MgCl_2,_ 7 mM CaEGTA, 6.99 mM Na_2_ATP, 15 mM Na_2_CrP, 40 µM leupeptin, 10 µM E64; pH 7.2) solutions were used during force measurements with a pCa (ie, -_10_log[Ca^2+^]) value of 10 and 4.5, respectively. Solutions with intermediate Ca^2+^ concentrations were prepared by mixing activating and relaxing solutions. Cardiomyocyte active force (F_active_) and calcium sensitivity of force production (characterized by pCa_50_) were determined by activations in solutions with different Ca^2+^ concentrations. Cardiomyocyte passive force (F_passive_) was measured in the relaxing solution.

### Determination of myofilament protein phosphorylation

A LV myocardial tissue sample (20 mg) was first handled similarly as for cardiomyocyte isolation, then homogenized in a sample buffer (containing 8 M urea, 2 M thiourea, 3% sodium dodecyl sulfate [SDS], 75 mM DTT, 0.05 M Tris-HCl [pH 6.8], 40 µM leupeptin, 10 µM E64, 10% glycerol, brome-phenol blue). After centrifugation (16 000 g for 5 minutes) protein concentration was measured from the supernatant with a dot-blot based method, where different dilutions from bovine serum albumine served as standard. Protein concentration of the samples was adjusted to 1 mg/mL. Polyacrylamide gels (2%, 4%, and 15% agarose-strengthened) were used to separate titin, MyBP-C, and TnI, respectively. Phosphorylation status of myofilament proteins was assessed by Pro-Q^®^ Diamond phosphoprotein staining (Invitrogen, Eugene, OR, USA), while protein composition was visualized by Coomassie blue (Reanal, Budapest, Hungary). Separation and identification of myofilament proteins were carried out based on their molecular weight and compared to those of a molecular weight standard (marker 4.6 kDa – 300 kDa).

### Data analysis and statistics

F_active_ and F_passive_ values were normalized to cardiomyocyte cross-sectional area and expressed in kN/m^2^. Calcium-force relationship was fitted by a modified Hill equation (42). Intensities of the protein bands were quantified by densitometry using the ImageJ 1.41o (NIH, Bethesda, MD, USA) and Magic Plot Student 2.5.1 (Saint Petersburg, Russia) softwares. Phosphorylation of myofilament proteins was normalized to the protein amount and expressed in percentages relative to an internal control (same LV rat sample on each gel). Statistical significance was calculated by analysis of variance (ANOVA followed by Bonferroni’s post hoc test) and linear regression. GraphPad Prism 5.0 (GraphPad Software Inc., San Diego, CA, USA) software was used. Values are given as mean ± standard error of the mean (SEM). The level of statistical significance was *P* < 0.05.

## Results

### Effects of ovariectomy and stress on basic parameters

Basic physical and laboratory parameters measured in the four animal groups are listed in Table 1. Both body weight and heart weight were significantly higher in the OVX-S group than in the control group, but heart-to-body weight ratios did not significantly differ. Serum basal glucose and total cholesterol levels were the same in all groups. Other relevant physiological parameters, such as heart rate and arterial blood pressure, were not assessed in this study.

**Table 1:** Basic physical and laboratory parameters measured in control, stressed control (control-S), ovariectomized (OVX), and stressed OVX (OVX-S) animals. Data are presented as mean ± standard error of the mean

	Control	Control-S	OVX	OVX-S	*P* value
Body weight (g)	288.4 ± 4.7	286.6 ± 2.4	297.8 ± 3.9	328.6 ± 5.8*^†‡^	<0.001
Heart weight (g)	1.1 ± 0.05	1.1 ± 0.03	1.2 ± 0.04	1.3 ± 0.04*	<0.050
Heart-to-body weight ratio	0.004 ± 0.0002	0.004 ± 0.0001	0.004 ± 0.0001	0.004 ± 0.0002	N.S.
Progesterone (ng/mL)	10 ± 2.9	35.6 ± 4.8*	2.8 ± 0.5	21.9 ± 4.0^‡^	<0.001*; <0.01^‡^
Glucose (mmol/L)	11.8 ± 0.9	9.7 ± 0.7	9.9 ± 0.4	10.3 ± 0.7	N.S.
Cholesterol (mmol/L)	2.4 ± 0.1	2.6 ± 0.3	2.5 ± 0.1	2.6 ± 0.1	N.S.

*Significant difference vs control.

†Significant difference vs control-S.

‡Significant difference vs OVX.

### Effects of ovariectomy and stress on serum estrogen and progesterone levels

**Figure 1:**
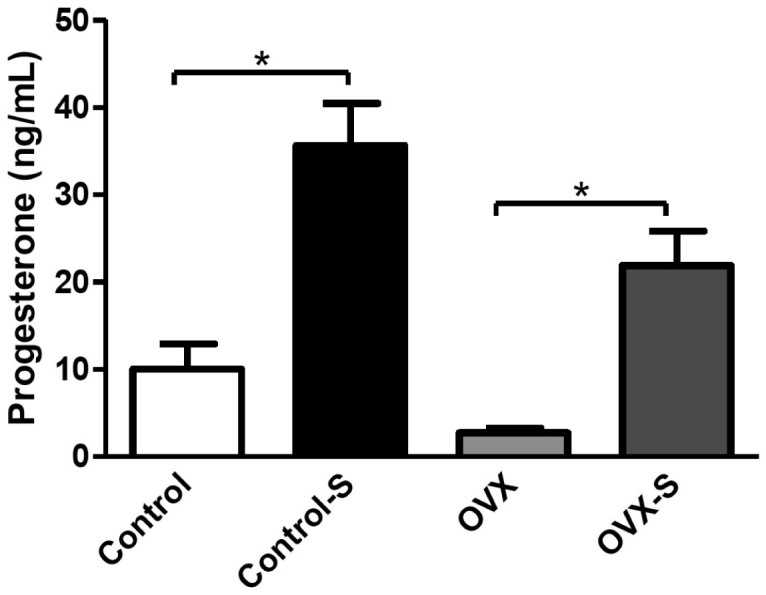
Serum progesterone levels measured using a chemiluminescent immunoassay in control and ovariectomized (OVX) rats not exposed and exposed to stress (control and OVX vs control-S and OVX-S, n = 8 for all groups). Bars are mean ± standard error of the mean and significant differences (*P* < 0.05) are denoted by asterisks.

Female sex hormones were measured from the sera of control and OVX animals. No measurable estrogen levels (detection limit was 4.9 pg/mL) were present in all OVX samples, indicating successful ovariectomy. Some of the control animals had measurable estrogen levels (about 25%), which was in accordance with the phases in the animal’s estrus cycle (data not shown). Progesterone levels were above the lower detection limit in all animal groups and were significantly increased in stressed control (35.6 ± 4.8 ng/mL) and stressed OVX (21.9 ± 4.0 ng/mL) rats compared to non-stressed control (10 ± 2.9 ng/mL) and OVX (2.8 ± 0.5 ng/mL) groups (n = 8 rats/group, *P* < 0.001 control-S vs control; *P* < 0.010 OVX-S vs OVX, Figure 1). The increase in progesterone levels during stress was comparable in control-S (25.6 ng/mL) and OVX-S (19.1 ng/mL) rats.

### Ovariectomy alters cardiomyocyte contractile function

**Figure 2:**
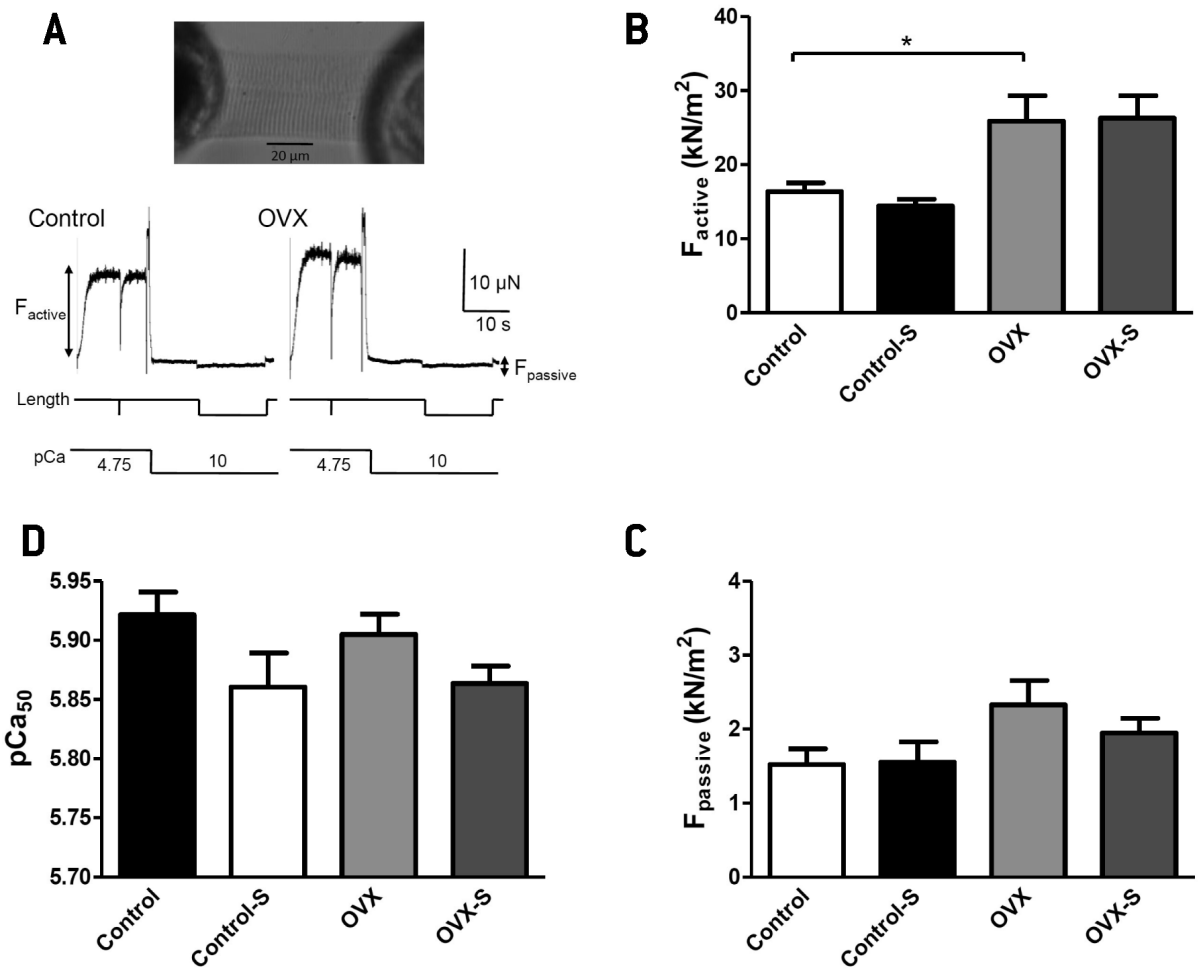
Effects of ovariectomy and stress on cardiomyocyte mechanics. (**A**) Single cardiomyocyte, isolated from rat myocardium, mounted between a sensitive force transducer and an electromagnetic motor (upper panel). Measurements of maximum (pCa [ie, -_10_log[Ca^2+^]] 4.75) Ca^2+^-dependent active (F_active_) and Ca^2+^-independent (pCa 10) passive (F_passive_) force levels in control and ovariectomized (OVX) animals (lower panel). (**B**) Effect of ovariectomy (OVX) and stress (control-S and OVX-S) on cardiomyocyte F_active_ (*P < 0.050 vs control). (**C**) Calcium sensitivity of force production (pCa_50_) determined in skinned cardiomyocytes derived from LV tissue in the four animal groups. (**D**) Unaltered F_passive_ by ovariectomy or stress (number of cardiomyocytes, n = 10 per group of 5-7 animals).

F_active_, F_passive,_ and calcium sensitivity of force production (pCa_50_) were determined in cardiomyocytes isolated from LV myocardium of rats at a sarcomere length of 2.3 µm (n = 10 cardiomyocytes per group) (Figure 2A). F_active_ was significantly higher in OVX and OVX-S animals than in control and control-S rats (25.9 ± 3.4 kN/m^2^ and 26.3 ± 3.0 kN/m^2^ vs 16.4 ± 1.2 kN/m^2^ and 14.4 ± 0.9 kN/m^2^, respectively, *P* < 0.050). However, no change in F_active_ was observed in control and OVX animals upon stress (Figure 2B). No significant differences in pCa_50_ were found between control and OVX animals, although a trend toward lower pCa_50_ was observed in control-S and OVX-S cardiomyocytes (Figure 2C). Neither ovariectomy nor stress altered F_passive_ in all groups (Figure 2D). There were no changes in cardiomyocyte structure after ovariectomy or stress (data not shown).

### Changes in myofilament protein composition and phosphorylation upon ovariectomy and stress

**Figure 3:**
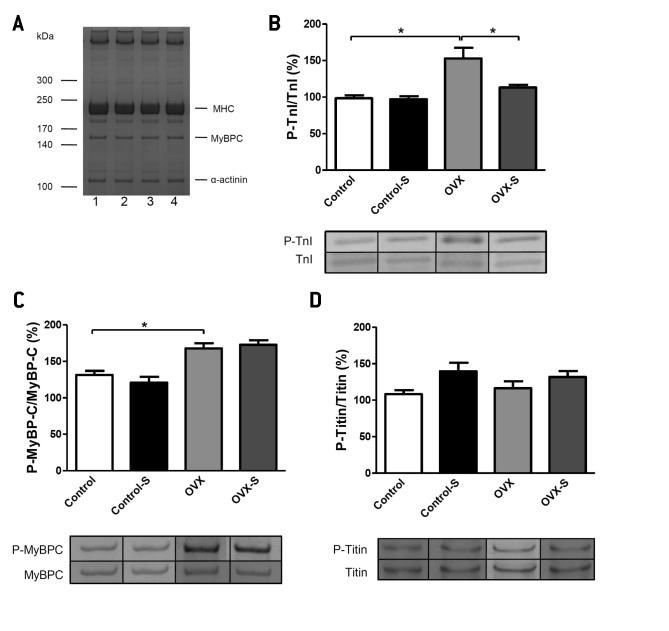
Representative examples of myofilament protein composition and protein phosphorylation in the left ventricular (LV) myocardium from control and ovariectomized (OVX) rats. (**A**) Myofilament protein composition in LV tissue homogenate in control – 1, stressed control (control-S) – 2, ovariectomized (OVX) – 3, and stressed-OVX (OVX-S) animals – 4 (Coomassie blue protein staining, MHC – myosin heavy chain). (**B**) Increased overall troponin I phosphorylation (P-TnI) in OVX, but not in OVX-S group (*P < 0.05 vs control). (**C**) Elevated myosin binding protein-C phosphorylation (P-MyBP-C) in OVX and OVX-S rats (*P < 0.05 vs control). (**D**) Similar titin phosphorylation (P-titin) in the four experimental groups (Phosphorylation of myofilament proteins was normalized to the protein amount and expressed in percentages relative to an internal control).

Myofilament protein composition was studied by SDS gel electrophoresis followed by quantitative protein staining. No considerable differences were detected in myocardial protein composition between control and OVX animals (Figure 3A). Using Pro-Q^®^ Diamond phosphoprotein staining, a significant increase in relative total TnI phosphorylation was observed in OVX animals compared to controls (153 ± 14.4% vs 98.5 ± 4%, *P* < 0.001). The increased TnI phosphorylation in OVX animals was not accompanied by a decrease in the pCa_50_ measured in the cardiomyocytes (r = 0.268, *P* = 0.732). Phosphorylation of TnI in OVX-S rats (113.1 ± 3.6%) was similar to the values measured in control and control-S animals (Figure 3B). Phosphorylation of MyBP-C was significantly higher in OVX rats than in controls (167.7 ± 7% vs 131.4 ± 5.5%, *P* < 0.001), but it remained unchanged under stress conditions in OVX-S rats (182.9 ± 6.2%, Figure 3C). In accordance with the unchanged F_passive_, phosphorylation of titin was the same in the four groups (Figure 3D).

### Correlation of cardiomyocyte function with myofilament phosphorylation and sex hormone levels

**Figure 4:**
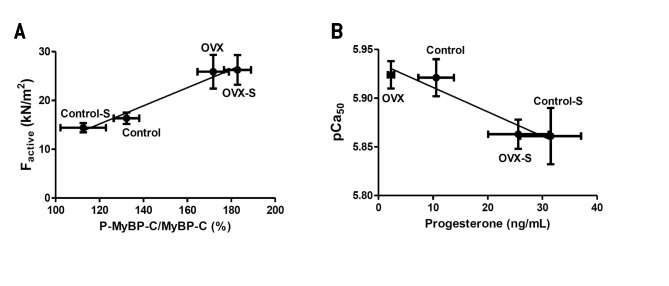
Correlations between myofilament phosphorylation, sex hormones, and cardiomyocyte function. (**A**) Strong correlation between myosin-binding protein C (MyBP-C) phosphorylation and cardiomyocyte F_active_ (r = 0.986, *P* < 0.05). (**B**) Calcium sensitivity of force production (pCa_50_) inversely correlated with serum progesterone levels (r = 0.963, *P* < 0.05). Correlation was assessed by fitting the values by a linear regression.

When phosphorylation of myofilament proteins and cardiomyocyte functional parameters were correlated, a strong positive correlation was observed between cardiomyocyte F_active_ and MyBP-C phosphorylation (r = 0.986, *P* < 0.050, Figure 4A). Titin phosphorylation did not correlate with cardiomyocyte F_passive_ (r = 0.139, *P* = 0.861). Serum progesterone levels and pCa_50_ of the cardiomyocytes measured in the same animals showed a significant inverse correlation (r = 0.963, *P* < 0.050, Figure 4B).

## Discussion

Several studies reported divergent effects of stress and female sex hormones on the cardiovascular system, however, their effect on cardiomyocyte contractile function is not fully elucidated. Our study showed that: I. serum progesterone levels were more than three times higher in control-S and OVX-S rats than in control and OVX animals; II. F_active_ of cardiomyocytes was significantly higher in OVX rats than in controls; III. F_passive_ and pCa_50_ was similar in OVX and control animals, although a moderate trend to lower pCa_50_ values was observed in control-S and OVX-S rats; IV. progesterone levels inversely correlated with pCa_50_ of cardiomyocytes; V. no major differences in myofilament protein composition were observed among the four groups, however, overall phosphorylation of TnI and MyBP-C was significantly higher in the OVX group than in controls.

Many studies showed that serum progesterone level increased under stress conditions due to its secretion from the adrenal cortex (20,21,23,43). In accordance with other findings (21), in the present study ovariectomy did not alter the amount of progesterone secreted during stress and the progesterone response under stress conditions.

Progesterone, as an intermediate product in the synthesis of cortisol, also has a crucial role in stress response (44) and in the regulation of the HPA axis (43). However, it is also important to note that acute and chronic stress have different effects on the activation of the sympathetic nervous system and HPA axis. Acute cold restraint stress applied in the present study has been shown to increase ACTH and corticosterone levels, which was followed by desensitization of the HPA response, to increase stroke volume and decrease the heart rate (45). Based on the presence of progesterone receptors within the myocardium (46), changes in progesterone levels could alter cardiomyocyte contractile properties. Indeed, the present study confirmed a significant correlation between serum progesterone levels and pCa_50_ of cardiomyocytes. This may indicate that the moderate decrease in pCa_50_ observed in control-S and OVX-S animals may be related to the increased progesterone levels. The inverse correlation between serum progesterone levels and myofilament Ca^2+^-sensitivity may provide a potential explanation for lowered cardiovascular risk in fertile women.

Experimental evidence confirms that ovariectomy induces up-regulation of cardiac β1-adrenergic receptor (β1-AR) expression, which can be prevented by estrogen and/or progesterone administration (47). β1-AR is a major regulator of the cardiac function and its activation increases heart rate and myocardial contractility (48). It was also found that ovariectomy increases the amplitude of the basal and isoprenaline-induced contractions, and induces an increase in β1-AR and a decrease in β2-AR expression (49). Stimulation of β1-AR activates G_s_ protein, leading to an increase in cAMP concentration. cAMP activates PKA, which phosphorylates myocardial proteins such as MyBP-C (50). Phosphorylation of MyBP-C accelerates cross-bridge kinetics (51) and increases calcium-activated maximal force production (52). In accordance with this observation, our study also revealed a significant increase in cardiomyocyte F_active_ in OVX rats. Moreover, phosphorylation of MyBP-C correlated with cardiomyocyte F_active_. Besides MyBP-C phosphorylation, changes in the distribution of the two myosin heavy chain (MHC) isoforms are also shown to be involved in the regulation of active force production (53). Due to limitations of myocardial tissue samples, in the present investigation we could not address this issue. Based on our findings, the increased F_active_ can be explained by the elevated phosphorylation of MyBP-C due to an increased β1-adrenerg stimulation after ovariectomy. This may indicate that the physiological regulatory effect of MyBP-C phosphorylation on cardiomyocyte active force generation is preserved after ovariectomy and stress.

Besides MyBP-C, phosphorylation of TnI and titin by PKA also alters cardiomyocyte contractile function, resulting in a decrease in pCa_50_ (29) and F_passive_ (54). In this study no major differences were found in pCa_50_ among the groups (only a moderate, non-significant decrease was observed in the control-S and OVX-S groups). Total phosphorylation of TnI, however, was increased in the OVX group, but this was not accompanied by a pCa_50_ decrease. This could result from the lack of specificity of the phosphoprotein staining for the different phosphorylation sites of the proteins. Therefore, despite the significant increase in total TnI phosphorylation, differences in the phosphorylation of the protein kinase-specific sites (PKA, PKC-βII, δ, ϵ) – which have been shown to have divergent effects on pCa_50_ (55) – cannot be excluded. A modulating effect of ovarian sex hormone deficiency on Ca^2+^-responsiveness of myofilament activation was demonstrated convincingly in the study by Wattanapermpool (27). In that study, an increase in the myofilament pCa_50_ and a decrease in the maximum myofibrillar ATPase activity was found in eight-week OVX rats at pH 7.0, together with an unchanged maximum ATPase activity and no differences in pCa_50_ at pH 6.5. However, when the study was extended to ten-week OVX rats, parallel to the suppression of maximum ATPase activities, a significant hypersensitivity of myofilaments to Ca^2+^ could also be detected at both pH 7.0 and pH 6.5 in the OVX group. This finding underlies the importance of the time and the progressive nature of myofilament alterations after ovariectomy. We did not detect significant difference in the myofilament Ca^2+^-sensitivity between control and OVX rats. 

In the present study, no correlation between cardiomyocyte F_passive_ and phosphorylation of its main regulator sarcomeric protein, titin was observed. This finding is consistent with an earlier report demonstrating unchanged passive stiffness in OVX animals (56). No differences were found in the phosphorylation of myofilament proteins between the control and control-S groups. The basal level of PKA-dependent protein phosphorylation in rodent hearts appears to be significantly higher than in large mammals (57). Accordingly, relative changes in the level of PKA-dependent protein phosphorylation under acute stress conditions and/or *in vitro* PKA challenges can be smaller in the hearts of small rodents than in large mammals. Moreover, sympathetic effects of acute stress tend to rapidly desensitize. Taking all this into consideration, we hypothesize that the combination of the above characteristics explains the unchanged phosphorylation level of myofilament proteins in control-S compared to control group.

Stress procedures used in this study induced physiological alterations and pathophysiological changes. However, the effects of this particular stress input (immobilization in combination with cold) may be unlikely in everyday human life. Further studies are necessary to confirm that the observed effects could be generalized for other types of acute stress.

Control animals were not sham-operated. Operation itself may have contributed to the differences between control and OVX groups. In future studies, sham-operated animals have to be used as controls. Type of anesthetics, in particular, application of isoflurane may have affected myocardial parameters (58). Also, the levels of estrogen and progesterone were low, considerably below the lower detection limit of the applied technique.

The present study assessed contractile properties of single, demembranated cardiomyocytes and focused mainly on alterations in the composition and phosphorylation of myofilament proteins. Changes in sarcolemmal, sarcoplasmic, and Ca^2+^-handling proteins cannot be studied under such experimental conditions as a result of the membrane solubilisation. Single cardiomyocytes may not represent overall myocardial function. Moreover, heart samples were frozen and their functional and biochemical properties were evaluated upon thawing. Nevertheless, this approach was supported by our previous experience, when we did not find significant effects of freezing and thawing on the functional properties of cardiomyocyte preparations independently of the species (30,59).

Single acute stress significantly increased serum progesterone levels in both control and OVX rats. Stress and female hormones altered cardiomyocyte contractile force generation possibly by increasing Ca^2+^-activated force production through myosin-binding protein C phosphorylation. 
